# Effect of low dose allopurinol on glycemic control and glycemic variability in patients with type 2 diabetes mellitus: A cross-sectional study

**DOI:** 10.1016/j.heliyon.2022.e11549

**Published:** 2022-11-09

**Authors:** Manal M. Alem

**Affiliations:** Department of Pharmacology, College of Clinical Pharmacy, Imam Abdulrahman Bin Faisal University, Dammam, Saudi Arabia

**Keywords:** Type 2 diabetes mellitus, Glycemic control, Glycated hemoglobin, Glycemic variability, Allopurinol, Xanthine oxidase

## Abstract

**Background:**

Type 2 diabetes mellitus (DM), gout, and asymptomatic hyperuricemia are inter-connected pathologies. Glycemic control (GC), involving a range of treatments is central to the management of DM, whereas allopurinol continues to be the most widely recommended urate lowering agent. Allopurinol has been shown to possess anti-oxidant properties: this study explores the potential effect of allopurinol on glucose homeostasis.

**Methods:**

This is an observational study with a cross-sectional design performed on patients with type 2 diabetes mellitus (DM), recruited from centers in Saudi Arabia. Patients were divided into two groups; allopurinol users; (for gout or asymptomatic hyperuricemia) and a matching disease control group. Patient demographics, co-morbid conditions, biochemical tests, and pharmacological treatments were extracted from electronic records to investigate the effect of allopurinol therapy on Glycemic control (GC), as assessed by glycated haemoglobin (HbA1c as primary endpoint), and on parameters of glycaemic variability (GV) (secondary endpoints).

**Results:**

A total of 194 patients with type 2 DM were recruited (97 in both groups). The two groups were matched for age, sex, and duration of DM: mean age: 59.4 years, 73% males, and 122 months in the allopurinol group vs 59.6 years, 73% males, and 113 months in the control group. Antidiabetic medications were matched between the two groups. In the allopurinol group, it was prescribed with a daily dose of 100 mg, for 77% of the patients, with median duration of 39.5 months. HbA1c values were; 6.90% (6.20, 7.80) in the allopurinol group vs 7.30% (6.60, 8.40) in the control group (P = 0.010). Parameters of GV were calculated from 3 consecutive fasting blood sugar (FBS) readings: variability independent of the mean (VIM) was 0.140 in the allopurinol group vs 0.987 in the control group (P < 0.001).

**Conclusion:**

Concomitant low-dose allopurinol therapy in patients with type 2 DM was associated with modest but significant improvements in GC and GV.

## Teaser

Type 2 diabetes mellitus; Glycemic control; Glycated hemoglobin; Glycemic variability; Allopurinol; Canthine oxidase.

## 1 Introduction

Type 2 diabetes mellitus (DM) has a global prevalence of 9.3% (2019), but it is expected to rise to 10.2% by 2030, and 10.9% by 2045 [1]. Such alarming numbers with the well-recognized macro- and micro-vascular complications constitute serious threats to the quality of life/life expectancy of affected patients. The UK Prospective Diabetes Study (UKPDS) has shown that glycemic control (GC) is the main therapeutic strategy to lower the risk of microvascular complications in newly diagnosed patients with type 2 diabetes [2]. A post-trial monitoring (10 years) has extended the benefits to include macrovascular complications. In summary, intensive therapy (in the sulfonylurea-insulin group) reduced the relative risk of microvascular disease by 24%, myocardial infarction by 15% and death from any cause by 13%. Correspondingly, in the metformin group, the relative risk reductions were 33% for myocardial infarction and 27% for death from any cause [3]. To achieve GC, detailed pharmacologic approaches have been described by the American Diabetes Association (ADA), issued as part of: *Standards of Medical Care in Diabetes 2021* [4, 5]*.*

Glycemic variability (GV) is a more recently identified phenomenon describing fluctuations or oscillations in blood glucose concentrations which can adversely affect outcomes [6]. GV can be classified as short-term GV (referring to within- or between days fluctuations) and long-term GV (referring to fluctuations over several weeks or months) [7]. These phenomena, independent of glycemic control, have been shown to be additional risks for diabetes-related complications; micro- and macro-vascular, and mortality [7]. In a post-hoc cohort analysis of data from the Antihypertensive and Lipid-Lowering treatment to prevent Heart Attack Trial (ALLHAT) that included patients with and without diabetes, it was shown that higher GV was associated with increased risk of all-cause mortality [8]. Similar findings have been reported by other studies confirming the association between GV and diabetic micro-, macro-vascular complications, and all-cause mortality [7, 9, 10].

The pathogenesis of diabetes, and its complications is multi-factorial and involves complex pathways including the inter-connection between diabetes, free radicals, endothelial function and diabetes-related complications [11]. Hyperglycemia has been shown to contribute to free radical generation, oxidative stress, endothelial dysfunction and, finally diabetes-related complications [12]. This series of events was discovered more than 30 years ago which introduced the concept that agents with antioxidant properties could be part of the therapeutic strategies in diabetes care [13].

One of the key enzyme systems involved in oxidative stress is xanthine oxidoreductase (XOR). This enzyme system exists in two forms; xanthine dehydrogenase (XDH) and xanthine oxidase (XO) [14]. The latter is a source of reactive oxygen species that contributes to oxidative stress in the diabetic state, where its activity was found to be significantly higher than in control subjects [15], and correlating positively with biomarkers of glycemic control (glycated hemoglobin, HBA1c) [15]. Such positive and significant correlation between XO activity and glycemic control has also been established in patients with multiple cardiovascular risk factors [16], and also in the general population [17, 18]. Further small-scale research studies have showed the benefits of allopurinol (≤300 mg/day) on GC, as expressed via the homeostatic model assessment of insulin resistance (HOMA-IR), in patients with asymptomatic hyperuricemia over treatment periods of 3 months [19], and 3 years [20], respectively.

This present observational study sought to extend these findings beyond GC to GV: to explore whether or not concomitant allopurinol therapy (as a xanthine oxidase inhibitor prescribed for gout or asymptomatic hyperuricemia) might have a beneficial effect on GC or GV in our local population with type 2 DM.

## 2 Methods

### 2.1 Study design and patients identification

**Figure 1:**
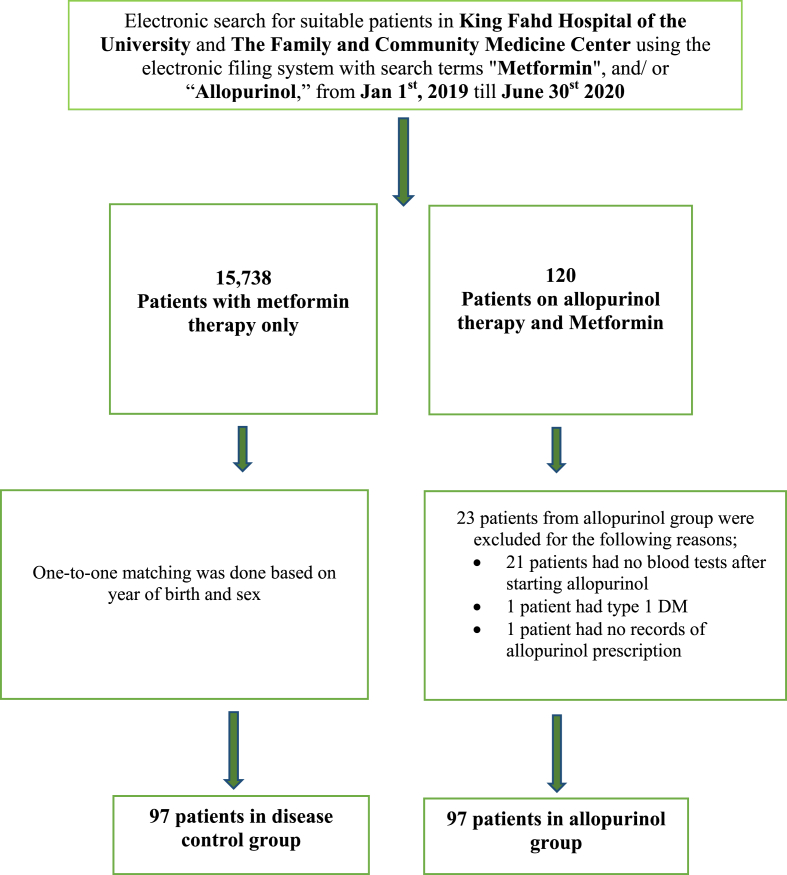
Study flow chart.

This analytical observation study was designed to study the association of concomitant allopurinol therapy on GC in patients with type 2 DM: HbA1c was the primary endpoint. The secondary endpoint was the corresponding association with GV. Eligible patients were identified from the diabetic centre and the general medical clinics of King Fahd Hospital of the University (KFHU), Al Khobar. In addition, patients were also recruited from the family and community medicine centre of Imam Abdulrahman bin Faisal University campus, Dammam. The cross-sectional design was selected as many patients were already receiving allopurinol therapy before referral to the recruitment sites. Patient search and identification were conducted by hospital pharmacy staff via access to the computerized filing system in KFHU, with straight numeric identification of each patient, primarily according to their prescribed medication (Figure 1). After identifying patients for the allopurinol group, the control patients were identified by one-to-one matching based on date of birth (year), and sex. The conduct and reporting of the study followed the recommended guidelines of the STROBE Statement: The Strengthening the Reporting of Observational Studies in Epidemiology [21].

### 2.2 Ethical approval and consent process

The study protocol was approved by the Institutional Review Board (IRB-2020-05-247), Deanship of Scientific Research, Imam Abdulrahman Bin Faisal University, Dammam, Saudi Arabia. Since the study does not involve any intervention, a waiver of informed consent from the participants was obtained. However, where data were incomplete (particularly in relation to the duration of allopurinol treatment), a protocol amendment was approved whereby informed verbal consent (via telephone conversation) was obtained for patients to provide such missing information. This consent process/protocol amendment was approved by the Institutional Review Board, Deanship of Scientific Research, Imam Abdulrahman Bin Faisal University, Dammam, Saudi Arabia. The study was conducted in accordance with the Declaration of Helsinki, the ICH Harmonized Tripartite Good Clinical Practice Guidelines, and the laws of Saudi Arabia.

### 2.3 Inclusion and exclusion criteria

**Inclusion criteria**

⁃ Age >18 years
⁃ Type 2 DM diagnosed using American Diabetes Association criteria and on pharmacological treatment (search terms- Figure 1)
⁃ Concomitant allopurinol therapy (with any dose) for the active arm only

**Exclusion criteria**

⁃ Type 1 DM
⁃ Active (known) malignant disease

### 2.4 Variables collected

The following patient demographic information was collected: reported co-morbid conditions, DM duration, DM medications, and other medications, outpatient reports and, where appropriate, details of allopurinol treatment (indication, start date and dose), presence/absence of complications e.g. renal stone disease. Classification of the patients based on estimated GFR (calculated by the abbreviated MDRD equation):

186 × (Creatinine/88.4)^−1.154^ × (Age)^−0.203^ × (0.742 if female) × (1.210 if black).

Biochemical variables were also collected, including HbA1c and matching 3 fasting blood sugar readings (collected over one year): from these measurements, GC and GV were calculated respectively. DM complications that might have occurred after study entry date were collected according to the definition of UKPDS; any macrovascular diabetes-related endpoint (sudden death, fatal/non-fatal myocardial infarction, angina, heart failure, stroke, renal failure, and amputation) and microvascular diabetes-related endpoint (vitreous hemorrhage, retinopathy, blindness, cataract, nephropathy and neuropathy) [2].

### 2.5 Statistical analysis

Baseline data are reported as mean ± Standard Deviation (SD) for continuous variables; number and percentages for categorical variables. Median and interquartile ranges were used for non-normally distributed data. Comparison of the two patients groups was by two-sample T-test and 95% confidence intervals (CI): the Mann-Whitney test and chi-square test where appropriate. Correlation analyses were performed using Spearman correlation coefficients (rho). The primary endpoint was the association of allopurinol use with HbA1c. Secondary endpoint was the association of allopurinol therapy with GV. All statistical analyses were performed using Minitab statistical software (version18, Minitab Inc., State College, PA, USA). A value of p < 0.05 was considered statistically significant.

### 2.6 Measures of glycemic variability

FBS readings, matched to the dates for HbA1c measurements, were available for 94 patients in the allopurinol group and 96 patients in the control group. Sufficient data were available to allow the calculation of dispersion or variability in 78 patients in the allopurinol group and 79 patients in the control group. Using the average of FBS readings, the calculation of visit-to-visit variability (VVV) of FBS was defined by more than one statistic. The first is the intra-individual SD across the visits; the second is the coefficient of variation (defined as the ratio of the SD/the mean FBS×100). These two statistics are correlated with the mean, tending to increase as the mean increases. A more appropriate statistic is the variability independent of the mean (VIM), that is calculated as SD×100/Mean^β^, where β is the regression coefficient based on natural logarithm of SD on natural logarithm of Mean FBS, and compared using Welch T-test [8].

### 2.7 Sample size

Using previous results [22], and assuming a pooled standard deviation of 2.05 units, the study would require a sample size of 89 patients in each group (a total sample size of 178 patients) to achieve a 90% power and a level of significance of 5% (two-sided), to detect a true difference of 1% in glycated hemoglobin means between allopurinol users, and control subjects.

## 3 Results

### 3.1 Demographic and clinical findings

The search process enabled the inclusion of 194 patients into the study, and 97 patients were in each group. Patients in the allopurinol group were matched to control group for age and sex, and for mean achieved blood pressure and heart rate: respectively, 133/79, 80 vs 132/79 mmHg, 80 beats/min. The allopurinol group had significantly higher body weight and body mass index (BMI) (estimated differences of, respectively, 7 kg (CI; 1, 13, P = 0.020) and 2.1 kg/m^2^ (CI; 0.2, 4, P = 0.029). The mean duration of DM was matched between the two groups; 122 months in the allopurinol group vs 113 months in the control group (P = 0.570). In terms of the co-morbid conditions, the two groups were well matched (see Table 1) apart from the prevalence of a history of systemic hypertension and chronic kidney disease which were significantly higher in allopurinol group; respectively, 85.6% vs 67% (P = 0.002) and 36.1% vs 7.2% (P < 0.001) (Table 1). In general, across the stages of chronic kidney disease (CKD), renal impairment was more prevalent in the allopurinol group. This included 12 patients in the allopurinol group (vs none in the control group) who were maintained on renal replacement therapy: 2 on hemodialysis and 10 on peritoneal dialysis.

**Table 1:** Demographic, and clinical comparisons between patients groups.

Parameter	Allopurinol group N = 97	Control group N = 97	P value
Age (years)	59.35 ± 13.18	59.56 ± 13.41	0.914
Sex (Males)	71 (73.20%)	71 (73.20%)	1.000
Height (cm)	165.05 ± 10.11	164.36 ± 8.43	0.609
Weight (Kg)	89.00 (71.50, 104.50)	82.00 (69.00, 94.00)	0.020
BMI	31.4 (28.05, 37.50)	30.20 (25.55, 34.15)	0.029
*Co-morbidities∗*			
Systemic Hypertension	83 (85.57%)	65 (67.01%)	0.002
Ischemic heart disease	27 (27.84%)	25 (25.77%)	0.746
Chronic heart failure	12 (12.37%)	7 (7.22%)	0.227
Cardiac arrhythmias/Atrial fibrillation	8 (8.25%)/6 (6.19%)	5 (5.15%)/3 (3.09%)	0.389/0.306
Transient ischemic attacks/Stroke	2 (2.06%)/7 (7.22%)	2 (2.06%)/13 (13.40%)	1.000/0.157
Chronic kidney disease	35 (36.08%)	7 (7.22%)	<0.001
Dyslipidemia	57 (58.76%)	53 (54.64%)	0.562
*Allopurinol indication∗∗*			-
Gout	34 (35.05%)	-	
Asymptomatic hyperuricemia	57 (58.76%)	-	
Renal stones (multiple/recurrent)	2 (2.06%)		
*Allopurinol therapy daily dose*			
100 (mg/day)	75 (77.32%)	-	
200 (mg/day)	7 (7.22%)	-	
300 (mg/day)	14 (14.43%)	-	
400 (mg/day)	1 (1.03%)	-	
*Allopurinol therapy duration (months)*	39.50 (10.95, 82.78)	-	
*Diabetes related biochemistry*			
Glycated hemoglobin (%)	6.90 (6.20, 7.80)	7.30 (6.60, 8.40)	0.010
Fasting blood sugar (mg/dL)∗∗∗	122.50 (105.88, 142.00)	131.67 (109.42, 166.50)	0.072
*Variability of FBS∗∗∗∗*			
Standard deviation (mean)	19.94	26.47	0.231
Coefficient of variation	14.03%	16.61%	0.256
Variability independent of the mean (VIM)	0.140	0.987	<0.001
*Renal biochemistry€*			
eGFR (ml/min/1.73m^2^)	70.07 ± 23.91	85.37 ± 28.52	<0.001
BUN (mg/dL)	17.00 (13.00, 23.00)	15.00 (11.00, 19.00)	0.005
Serum creatinine (mg/dL)	1.12 (0.90, 1.41)	0.93 (0.78, 1.15)	<0.001
Uric acid (mg/dL)¥	6.45 ± 1.78	5.18 ± 1.51	<0.001

*Abbreviations*: BMI: body mass index; FBS: fasting blood sugar; eGFR: estimated glomerular filtration rate; BUN: blood urea nitrogen.

Data ∗ was inclusive of probable 6 Pre-DM in allopurinol group vs 1 Pre-DM in control group (P = 0.054).

Data ∗∗ was based on 93 patients; 4 patients had no mentioned indication. 2 patients from those who had gout and 3 from those labelled with asymptomatic hyperuricemia had also multiple/recurrent renal stones.

Data ∗∗∗ based on 94 patients in allopurinol group, and 96 patients in control group.

Data ∗∗∗∗ based on 78 patients in allopurinol group, and 79 patients in control group.

Data€ based on 84 patients in allopurinol group; one patient did not have matching renal function tests, and 12 were on renal replacement therapy, and 97 patients in control group.

Data ¥ based on 90 patients in allopurinol group, and 34 patients in control group.

### 3.2 Allopurinol indications and dose

Allopurinol therapy with indication, dose, and duration (in months before HbA1c readings) are listed in Table 1. The most frequent dose was 100 mg/day in 77.3% of the whole group, while the median duration for allopurinol therapy was 39.5 months (10.95, 82.78).

### 3.3 Comparisons of parameters of glycemic control (GC)

HbA1c readings comparison between the two groups showed a small yet significant difference in favor of allopurinol therapy. The estimated difference was −0.5%, (CI: −0.8, −0.1) (P = 0.010) (Table 1).

### 3.4 Sensitivity analysis

Advanced CKD is associated with shortened red blood cells lifespan which might reduce HbA1c readings. Accordingly, the HbA1c comparison was repeated after removal of the 12 patients on renal replacement therapy (85 allopurinol patients, vs 97 control patients). The estimated difference obtained for HbA1c was −0.4%, (CI: −0.7, −0.0) (P = 0.033). Since the study population might have included probable Pre-DM patients (6 in the allopurinol group, and 1 in control group), HbA1c comparison was repeated after removal of these patients (91 allopurinol patients, vs 96 control patients). The estimated difference obtained for HbA1c was −0.4%, (CI: −0.8, −0.0) (P = 0.030) in favor of allopurinol therapy.

### 3.5 Comparisons of parameters of glycemic variability (GV)

**Figure 2:**
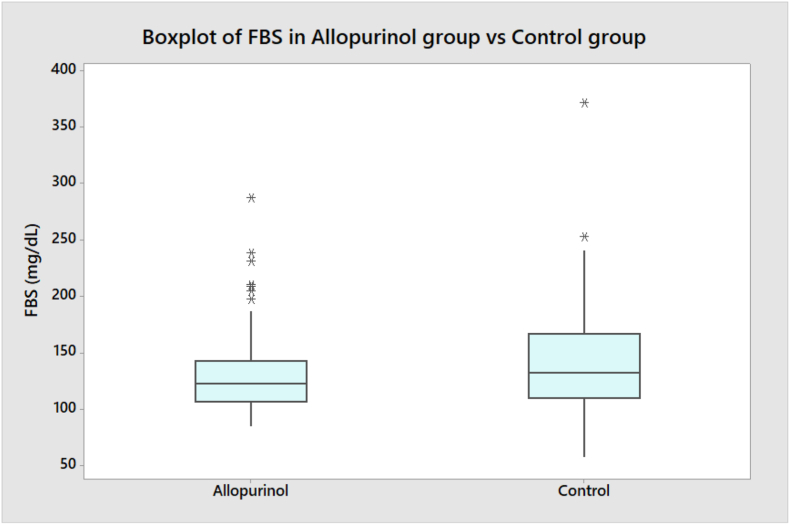
Comparison of FBS reading in both patients groups.
• Comparisons of standard deviation, and coefficient of variation showed a trend for less dispersion in FBS readings in allopurinol users (Table 1).
• Comparisons of the VIM showed a statistically significant results in favor of allopurinol therapy (P < 0.001) (Table 1 and Figure 3).

• Comparing FBS readings between the two groups is demonstrated in (Table 1) as medians with interquartile ranges (Q1, Q3) with a trend towards lower reading in favor of allopurinol users, with an estimated difference of −8 mg/dL (CI: −17.33, 1) (P = 0.072).
• Comparing the range of FBS readings with the minimum and maximum values were; 84.33, and 287.00 mg/dL in the allopurinol group, as compared with 57.00, and 372.00 mg/dL in the control group (see Figure 2).

**Figure 3:**
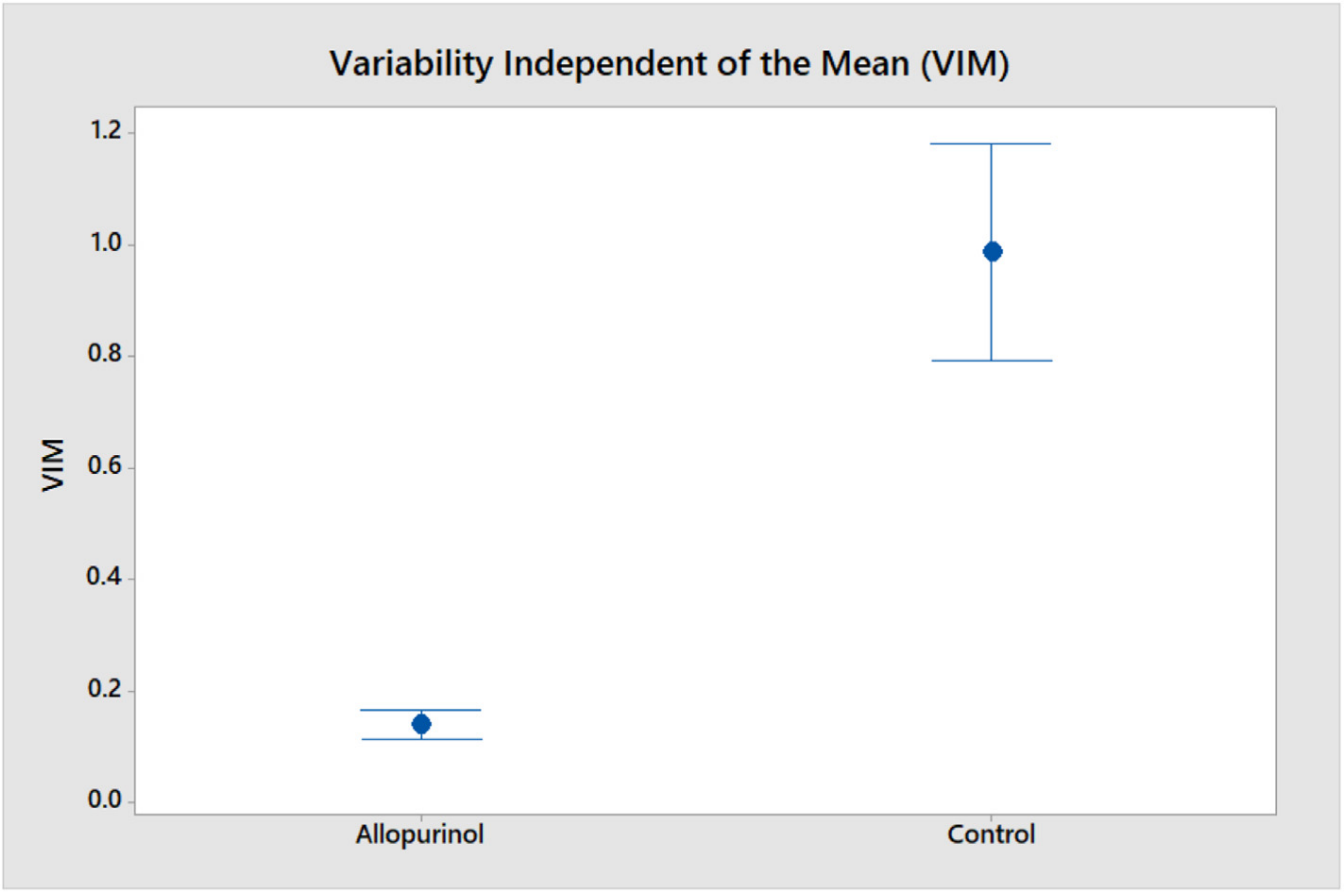
Comparison of VIM reading in both patients groups.

### 3.6 Correlation between GC and GV indices

By studying the correlation between two parameters of glycemic control; HbA1c, and FBS, with an index of GV; VIM, correlation analysis revealed that FBS was not correlated with VIM in both groups; Spearman correlation coefficients (rho); (r = −0.018, P = 0.876) in allopurinol group, and (r = 0.027, P = 0.816) in control group. While HbA1c had no correlation with VIM in allopurinol group (r = 0.044, P = 0.701), it had a significant positive correlation in control group (r = 0.273, P = 0.015).

### 3.7 Comparisons of other biochemical parameters

As expected, the renal biochemical parameters (blood urea nitrogen, serum creatinine and serum uric acid concentrations) were higher in the allopurinol group (Table 1). There were no clinically relevant differences for sodium, potassium or chloride.

### 3.8 Comparisons of pharmacological therapy

The two groups were matched in terms of antidiabetic medications (Table 2), but they differed slightly in the use of angiotensin receptor blockers (52.6% in the allopurinol group vs 36.1% in the control group), beta blockers and calcium channel blockers (49.5% vs 27.8%). There also were small differences for diuretic and aspirin use (Table 2).

**Table 2:** Therapeutics comparisons between patients groups.

Parameter	Allopurinol group N = 97	Control group N = 97	P value
*Diabetes mellitus medications*			
Insulin	25 (25.77%)	27 (27.94%)	0.746
Sulphonylureas	14 (14.43%)	20 (20.62%)	0.257
Metformin	89 (91.75%)	94 (96.91%)	0.121
Dipeptidyl peptidase-4 (DPP-4) inhibitors	30 (30.93%)	35 (36.08%)	0.447
Sodium Glucose cotransporters (SGLTs) inhibitors	9 (9.28%)	8 (8.25%)	0.800
Glucagon-like peptide-1 (GLP-1) agonists	6 (6.19%)	1 (1.03%)	0.054
*Other medications*			
Angiotensin Converting Enzyme Inhibitors (ACEI)/ Angiotensin Receptor Blockers (ARB)	16 (16.49%)/51 (52.58%)	24 (24.74%)/35 (36.08%)	0.156/0.021
Beta-blockers/Calcium channel blockers	48 (49.48%)/48 (49.48%)	27 (27.84%)/27 (27.84%)	0.002/0.002
Hydrochlorothiazide/Furosemide	26 (26.80%)/22 (22.68%)	15 (15.46%)/10 (10.31%)	0.053/0.020
Spironolactone	8 (8.25%)	3 (3.09%)	0.121
Aspirin/Clopidogrel	44 (45.36%)/8 (8.25%)	58 (59.79%)/10 (10.31%)	0.044/0.621
Statins/Fenofibrate	78 (80.41%)/10 (10.31%)	77 (79.38%)/7 (7.22%)	0.858/0.446
Nitrates	8 (8.25%)	7 (7.22%)	0.788
Digoxin	1 (1.03%)	1 (1.03%)	1.000
Warfarin	1 (1.03%)	2 (2.06%)	0.561

Chi-squared test for association was used for the comparisons.

### 3.9 Diabetes complications

After a mean follow up period of 43 ± 19 months, 17 patients had macrovascular complications in the allopurinol group (8 coronary artery disease, 6 diabetes-related mortality, 3 heart failure, and 3 stroke) as compared with 21 patients in the control group (11 coronary artery disease, 5 diabetes-related mortality, 2 heart failure, and 6 stroke) (P = 0.469). 10 patients had microvascular complications in the allopurinol group (5 retinopathy, 4 nephropathy, 3 neuropathy, and 1 cataract) as compared with 13 patients in the control group (3 retinopathy, 2 nephropathy, 4 neuropathy, and 4 cataract) (P = 0.505).

## 4 Discussion

In this observational study, it has been shown that low dose allopurinol therapy in patients with type 2 diabetes mellitus was associated with modest, yet significant effect on GC, as assessed by the most widely used biochemical marker, HbA1c. In addition, such therapy was also associated with a significant effect on GV, as assessed by a parameter statistically independent from the mean FBS values (variability independent of the mean). The following are the immediate and obvious questions:

• Is the magnitude of the improvement in GC of clinical significance?
• Is the magnitude of the improvement in GV of clinical significance?
• Do these two parameters/phenomena correlate?

Improvement in GC: what is a clinically significant drop in HbA1c?

The UKPDS has shown that 1% reduction in HbA1c was associated with risk reduction of 21% for any endpoint related to diabetes; 21% for death related to diabetes; 14%for myocardial infarction and 37% for microvascular complications [23]. The study emphasized also that, due to the lack of thresholds of glycemia for those complications, any reduction in HbA1c would be likely to reduce the risk of these complications, especially if the concentration approached the normal range [23]. Despite differences in study design, careful comparisons of the reduction achieved in this “observational study” (−0.5% with allopurinol) with the effects of established antidiabetic drug groups assessed via “randomized controlled trials” in patients with type 2 DM but with sub-optimal control are as follows. Glucagon-like peptide 1 (GLP-1) agonists (e.g. exenatide, liraglutide, and dulaglutide) have been shown to achieve −0.55 to −1.38 % reduction in HbA1c [24,25]; dipeptidyl peptidase 4 (DDP 4) inhibitors (e.g. vildagliptin and sitagliptin) can achieve −0.73, and −0.74% reduction, respectively [26]; sodium-glucose co-transporter 2 (SGLT 2) inhibitors (e.g. dapagliflozin) can achieve −0.50 to 0.66% reduction [27, 28, 29], while insulin glargine can achieve −0.9 to −1.11% reduction [30, 31]. Accordingly, the reduction achieved by low-dose allopurinol therapy in this study appears to be clinically significant.

A further consideration is that patients in the allopurinol group had higher BMI, higher prevalence of hypertension, more pronounced CKD, and a higher percentage of use of ARBs, CCBs, beta-blockers, and diuretics. These comorbidities, along with beta-blockers and diuretics, are known to have negative effects on glucose homeostasis [32], raising the possibility of an otherwise greater beneficial effect from allopurinol. In that regard, the magnitude of drop in HbA1c in this study was smaller than was observed in another sample of our population in a previous study of similar sample size, allopurinol dose and duration (−0.8% reduction) [22].

Regarding, GV: there is lack of agreement in the literature on the parameter that best reflects GV; there also is a lack of agreement on how to derive the relevant parameters. For example, one recent study assessed the prognostic significance of GV calculated from HbA1c vs that calculated from FBS and found that GV parameters calculated from FBS readings were more consistent with the metabolic outcomes and complications [10]. There also are statistical considerations and methodological considerations [33]. However, in an attempt to relate the results obtained in this study to a reference method, the ALLHAT study suggested that VIM is possibly the most robust measure of GV insofar as each unit change in VIM was associated with a higher risk of death; HR 1.014 (95% CI 1.006–1.022) (P = 0.001) [8]. While allopurinol benefits on insulin resistance were demonstrated previously [19, 20], our study is the first, to the best of our knowledge, to demonstrate an additional benefit on GV. DM complications reported in the allopurinol group were slightly fewer than those in the control group. However, this difference did not reach a statistical significance, presumably an issue of statistical power.

With regard to the relationship between GC and GV, the findings of this study identify no direct or definite relationship. This is in agreement with those few studies in the literature that have assessed both phenomena simultaneously using mean amplitude of glycemic excursions (MAGE), calculated over 48–72 hours over continuous sub-cutaneous interstitial glucose monitoring (short-term GV). Monnier *et al.* demonstrated that markers of oxidative stress were higher in type 2 DM patients in comparison with control subjects, and they correlated strongly with GV estimated by MAGE, and not with GC estimated by HbA1c [34]. Another two studies by Rizzo *et al.* and by Kim *et al.* compared vildagliptin; a DDP 4 inhibitors vs sitagliptin in the first, and vs pioglitazone in the second. Both studies showed that the two arms had a similar effect on GC (HbA1c), however, only vildagliptin had an extra advantage on GV assessed by (MAGE) [35, 36]. Another important finding was that such reduction in GV was associated with reduction of markers of oxidative stress and systemic inflammation, an association that was not found to exist with GC indices [35]. According to the current evidence, and the correlation analysis in this study, both GC and GV can co-exist in patients but it is likely that different pathways are involved and differential responses to pharmacological intervention.

Finally, the inter-relationships between glucose homeostasis and urate metabolism. So-called asymptomatic hyperuricemia, as a persistent biochemical abnormality, not only predisposes to gout but is also an independent risk factor for type 2 DM [37, 38]. The interaction between serum uric acid with insulin concentration and glucose homeostasis is complex and beyond the scope of our study. Number of studies that assessed allopurinol effect on glycemic control are few (with none so far to extend to glycemic variability). Recent meta-analysis have shown that allopurinol reduced significantly FBS, and showed a trend for HbA1c reduction [39]. However the dose that was considered effective was ≥200 mg daily [39]. Another two studies in type 2 DM patients contrastingly have shown negative results with 100 mg daily dose taken for 4 months [40], and 300 mg daily dose taken for 2 weeks [41].

The mechanism by which allopurinol produced the benefits observed in this study was not investigated. Oxidative stress plays an essential role in the pathogenesis of type 2 DM and its complications [42]. Several antioxidants have shown some benefits in type 2 DM patients and animal models of diabetes, among which allopurinol was considered as a promising agent [43]. Allopurinol has shown its ability to ameliorate inflammation in type 2 DM patients [20] and animal models [44], and it has shown that it upregulates adiponectin receptors 1 & 2, heme oxygenase-1 expression, and endothelial nitric oxide synthase (eNOS) expression in animal models [45]. Thus its mechanism might involve alteration of oxidative stress and inflammation, and such effects were found to persist for 2 years [46].

Allopurinol, has shown benefits in cardiovascular outcomes in type 2 DM patients [47] and other population [48]. Perhaps these observations can be attributed to a favorable metabolic profile “at least partially”: this might encourage health care professionals to lower the threshold for the prescription of allopurinol.

## 5 Limitations

This study has a number of limitations. Firstly, the cross-sectional design was selected as it was not feasible to obtain glycemic control indices in all patients prior to the commencement of allopurinol. Although the observational design does not allow the definitive identification of a “cause and effect relationship”, it is recognized as a method of “hypothesis generation”: the hypothesis can then be tested by a more robust study design. There also were differences between the groups in relation to renal function: assessing glycemic control by glycated hemoglobin may be less reliable in patients with advanced CKD. Other glycated proteins “that are still under research” were not routinely available. Other differences between the two groups (BMI and incidence of hypertension) would be expected to be associated with gout/hyperuricemia. Finally, the concept of glycemic variability is still new in the literature; there is still no agreement/consensus on the best index of GV.

## 6 Conclusion

In this observational study, it has been shown that low dose allopurinol therapy in patients with type 2 DM was associated with a favorable impact on glycemic control. Although the benefit was of a lesser magnitude, it is expected to be of a clinical significance if confirmed by further testing. In addition, allopurinol use was associated with an additional favorable effect on long-term GV, a new risk factor that has been shown to be independently associated with DM complications. Allopurinol is an old drug with an established safety record, no association with hypoglycemia and with low cost. Accordingly, future research is warranted to determine whether or not allopurinol has beneficial effects not only on glucose homeostasis but also on the micro- and macro-vascular complications in patients with type 2 DM.

## Declarations

### Author contribution statement

Manal M. Alem: Conceived and designed the experiments; Performed the experiments; Analyzed and interpreted the data; Contributed materials, analysis tools or data; Wrote the paper.

### Funding statement

This research did not receive any specific grant from funding agencies in the public, commercial, or not-for-profit sectors.

### Data availability statement

Data will be made available on request.

### Declaration of interest’s statement

The authors declare no conflict of interest.

### Additional information

No additional information is available for this paper.

## References

[1] Saeedi P., Petersohn I., Salpea P., Malanda B., Karuranga S., Unwin N. (2019).

[2] (1998). Intensive blood-glucose control with sulphonylureas or insulin compared with conventional treatment and risk of complications in patients with type 2 diabetes (UKPDS 33). UK Prospective Diabetes Study (UKPDS) Group. Lancet.

[3] Holman R.R., Paul S.K., Bethel M.A., Matthews D.R., Neil H.A. (2008). 10-year follow-up of intensive glucose control in type 2 diabetes. N. Engl. J. Med..

[4] (2021). 9. Pharmacologic approaches to glycemic treatment: <em>Standards of medical care in diabetes—2021</em>. Diabetes Care.

[5] American Diabetes A. (2021). 6. Glycemic targets: standards of medical care in diabetes-2021. Diabetes Care.

[6] Ceriello A., Esposito K., Piconi L., Ihnat M.A., Thorpe J.E., Testa R. (2008). Oscillating glucose is more deleterious to endothelial function and oxidative stress than mean glucose in normal and type 2 diabetic patients. Diabetes.

[7] Gorst C., Kwok C.S., Aslam S., Buchan I., Kontopantelis E., Myint P.K. (2015). Long-term glycemic variability and risk of adverse outcomes: a systematic review and meta-analysis. Diabetes Care.

[8] Echouffo-Tcheugui J.B., Zhao S., Brock G., Matsouaka R.A., Kline D., Joseph J.J. (2019). Visit-to-Visit glycemic variability and risks of cardiovascular events and all-cause mortality: the ALLHAT study. Diabetes Care.

[9] Zinman B., Marso S.P., Poulter N.R., Emerson S.S., Pieber T.R., Pratley R.E. (2018). Day-to-day fasting glycaemic variability in DEVOTE: associations with severe hypoglycaemia and cardiovascular outcomes (DEVOTE 2). Diabetologia.

[10] Slieker R.C., van der Heijden A., Nijpels G., Elders P.J.M., t Hart L.M., Beulens J.W.J. (2019). Visit-to-visit variability of glycemia and vascular complications: the Hoorn Diabetes Care System cohort. Cardiovasc. Diabetol..

[11] Maritim A.C., Sanders R.A., Watkins J.B. (2003). Diabetes, oxidative stress, and antioxidants: a review. J. Biochem. Mol. Toxicol..

[12] Ceriello A. (2006). Oxidative stress and diabetes-associated complications. Endocr. Pract..

[13] Hunt J.V., Dean R.T., Wolff S.P. (1988). Hydroxyl radical production and autoxidative glycosylation. Glucose autoxidation as the cause of protein damage in the experimental glycation model of diabetes mellitus and ageing. Biochem. J..

[14] Nishino T., Okamoto K. (2015). Mechanistic insights into xanthine oxidoreductase from development studies of candidate drugs to treat hyperuricemia and gout. J. Biol. Inorg. Chem..

[15] Kuppusamy U.R., Indran M., Rokiah P. (2005). Glycaemic control in relation to xanthine oxidase and antioxidant indices in Malaysian Type 2 diabetes patients. Diabet. Med..

[16] Fujimura Y., Yamauchi Y., Murase T., Nakamura T., Fujita S.I., Fujisaka T. (2017). Relationship between plasma xanthine oxidoreductase activity and left ventricular ejection fraction and hypertrophy among cardiac patients. PLoS One.

[17] Furuhashi M., Matsumoto M., Tanaka M., Moniwa N., Murase T., Nakamura T. (2018). Plasma xanthine oxidoreductase activity as a novel biomarker of metabolic disorders in a general population. Circ. J..

[18] Washio K.W., Kusunoki Y., Murase T., Nakamura T., Osugi K., Ohigashi M. (2017). Xanthine oxidoreductase activity is correlated with insulin resistance and subclinical inflammation in young humans. Metabolism.

[19] Takir M., Kostek O., Ozkok A., Elcioglu O.C., Bakan A., Erek A. (2015). Lowering uric acid with allopurinol improves insulin resistance and systemic inflammation in asymptomatic hyperuricemia. J. Invest. Med..

[20] Liu P., Wang H., Zhang F., Chen Y., Wang D., Wang Y. (2015). The effects of allopurinol on the carotid intima-media thickness in patients with type 2 diabetes and asymptomatic hyperuricemia: a three-year randomized parallel-controlled study. Intern. Med..

[21] von Elm E., Altman D.G., Egger M., Pocock S.J., Gotzsche P.C., Vandenbroucke J.P. (2007). The Strengthening the Reporting of Observational Studies in Epidemiology (STROBE) statement: guidelines for reporting observational studies. PLoS Med..

[22] Alem M.M., Aldosari S.R., Alkahmous A.A., Obad A.S., Fagir N.M., Al-Ghamdi B.S. (2019). Effect of long-term allopurinol therapy on left ventricular mass index in patients with ischemic heart disease; A cross-sectional study. Vasc. Health Risk Manag..

[23] Stratton I.M., Adler A.I., Neil H.A., Matthews D.R., Manley S.E., Cull C.A. (2000). Association of glycaemia with macrovascular and microvascular complications of type 2 diabetes (UKPDS 35): prospective observational study. BMJ.

[24] Htike Z.Z., Zaccardi F., Papamargaritis D., Webb D.R., Khunti K., Davies M.J. (2017). Efficacy and safety of glucagon-like peptide-1 receptor agonists in type 2 diabetes: a systematic review and mixed-treatment comparison analysis. Diabetes Obes. Metabol..

[25] Andreadis P., Karagiannis T., Malandris K., Avgerinos I., Liakos A., Manolopoulos A. (2018). Semaglutide for type 2 diabetes mellitus: a systematic review and meta-analysis. Diabetes Obes. Metabol..

[26] Amori R.E., Lau J., Pittas A.G. (2007). Efficacy and safety of incretin therapy in type 2 diabetes: systematic review and meta-analysis. JAMA.

[27] Liu X.Y., Zhang N., Chen R., Zhao J.G., Yu P. (2015). Efficacy and safety of sodium-glucose cotransporter 2 inhibitors in type 2 diabetes: a meta-analysis of randomized controlled trials for 1 to 2 years. J. Diabet. Complicat..

[28] Musso G., Gambino R., Cassader M., Pagano G. (2012). A novel approach to control hyperglycemia in type 2 diabetes: sodium glucose co-transport (SGLT) inhibitors: systematic review and meta-analysis of randomized trials. Ann. Med..

[29] Vasilakou D., Karagiannis T., Athanasiadou E., Mainou M., Liakos A., Bekiari E. (2013). Sodium-glucose cotransporter 2 inhibitors for type 2 diabetes: a systematic review and meta-analysis. Ann. Intern. Med..

[30] Marso S.P., McGuire D.K., Zinman B., Poulter N.R., Emerson S.S., Pieber T.R. (2017). Efficacy and safety of degludec versus glargine in type 2 diabetes. N. Engl. J. Med..

[31] Heine R.J., Van Gaal L.F., Johns D., Mihm M.J., Widel M.H., Brodows R.G. (2005). Exenatide versus insulin glargine in patients with suboptimally controlled type 2 diabetes: a randomized trial. Ann. Intern. Med..

[32] Rizos C.V., Elisaf M.S. (2014). Antihypertensive drugs and glucose metabolism. World J. Cardiol..

[33] Lee S., Lee H., Kim Y., Kim E. (2019). Effect of DPP-IV inhibitors on glycemic variability in patients with T2DM: a systematic review and meta-analysis. Sci. Rep..

[34] Monnier L., Mas E., Ginet C., Michel F., Villon L., Cristol J.P. (2006). Activation of oxidative stress by acute glucose fluctuations compared with sustained chronic hyperglycemia in patients with type 2 diabetes. JAMA.

[35] Rizzo M.R., Barbieri M., Marfella R., Paolisso G. (2012). Reduction of oxidative stress and inflammation by blunting daily acute glucose fluctuations in patients with type 2 diabetes: role of dipeptidyl peptidase-IV inhibition. Diabetes Care.

[36] Kim N.H., Kim D.L., Kim K.J., Kim N.H., Choi K.M., Baik S.H. (2017). Effects of vildagliptin or pioglitazone on glycemic variability and oxidative stress in patients with type 2 diabetes inadequately controlled with metformin monotherapy: a 16-week, randomised, open label, pilot study. Endocrinol. Metab. (Seoul).

[37] Bhole V., Choi J.W., Kim S.W., de Vera M., Choi H. (2010). Serum uric acid levels and the risk of type 2 diabetes: a prospective study. Am. J. Med..

[38] Dehghan A., van Hoek M., Sijbrands E.J., Hofman A., Witteman J.C. (2008). High serum uric acid as a novel risk factor for type 2 diabetes. Diabetes Care.

[39] Chen J., Ge J., Zha M., Miao J.-J., Sun Z.-L., Yu J.-Y. (2020). Effects of uric acid-lowering treatment on glycemia: a systematic review and meta-analysis. Front. Endocrinol..

[40] Momeni A., Shahidi S., Seirafian S., Taheri S., Kheiri S. (2010). Effect of allopurinol in decreasing proteinuria in type 2 diabetic patients. Iran J. Kidney Dis..

[41] Afshari M., Larijani B., Rezaie A., Mojtahedi A., Zamani M.J., Astanehi-Asghari F. (2004). Ineffectiveness of allopurinol in reduction of oxidative stress in diabetic patients; a randomized, double-blind placebo-controlled clinical trial. Biomed. Pharmacother..

[42] Singh A., Kukreti R., Saso L., Kukreti S. (2022). Mechanistic insight into oxidative stress-triggered signaling pathways and type 2 diabetes. Molecules.

[43] Rahimi R., Nikfar S., Larijani B., Abdollahi M. (2005). A review on the role of antioxidants in the management of diabetes and its complications. Biomed. Pharmacother..

[44] El-Bassossy H.M., Elberry A.A., Azhar A., Ghareib S.A., Alahdal A.M. (2015). Ameliorative effect of allopurinol on vascular complications of insulin resistance. J. Diabetes Res..

[45] Mostafa-Hedeab G., Shahataa M., Fouaad Ali E., Sabry Dina, EL-Nahass EL-Shaymaa, Hassan Manal, Mahmoud Fatma (2017). Allopurinol ameliorates high fructose diet-induced metabolic syndrome via up-regulation of adiponectin receptors and heme oxygenase-1 expressions in rats. Biomed. Pharmacol. J..

[46] Huang Y., Zhang C., Xu Z., Shen J., Zhang X., Du H. (2017). Clinical Study on efficacy of allopurinol in patients with acute coronary syndrome and its functional mechanism. Hellenic J. Cardiol..

[47] Weisman A., Tomlinson G.A., Lipscombe L.L., Perkins B.A., Hawker G.A. (2019). Association between allopurinol and cardiovascular outcomes and all-cause mortality in diabetes: a retrospective, population-based cohort study. Diabetes Obes. Metabol..

[48] Bredemeier M., Lopes L.M., Eisenreich M.A., Hickmann S., Bongiorno G.K., d’Avila R. (2018). Xanthine oxidase inhibitors for prevention of cardiovascular events: a systematic review and meta-analysis of randomized controlled trials. BMC Cardiovasc. Disord..

